# Renal microcirculation in acute kidney injury: bridging haemodynamics, inflammation and repair

**DOI:** 10.1016/j.aicoj.2026.100169

**Published:** 2026-09-23

**Authors:** Stanislas Abrard, John R. Prowle, Philippe Guerci, Didier Payen, Thomas Rimmelé

**Affiliations:** a Department of Anesthesiology and Critical Care Medicine, Hospices Civils de Lyon, Edouard Herriot Hospital, Lyon, France; b Research on Healthcare Performance RESHAPE, INSERM U1290, Université Claude Bernard Lyon 1, Lyon, France; c Critical Care and Peri-Operative Medicine Research Group, Faculty of Medicine and Dentistry, William Harvey Research Institute, Queen Mary University of London, London, United Kingdom; d Adult Critical Care Unit, The Royal London Hospital, Barts Health NHS Trust, Whitechapel Road, London, United Kingdom; e Department of Anesthesiology and Intensive Care, University Hospital of Nancy, Rue du Morvan, 54500 Vandoeuvre-lès-Nancy, France; f INSERM 1116, Acute and Chronic Heart Failure, University of Lorraine, Campus Brabois-Santé, 9 Avenue de la Forêt de Haye, 54500 Vandoeuvre-lès-Nancy, France; g Université Côte d’Azur, CHU de Nice, INSERM, U1065, C3M, Nice, France; h Service Maladies Infectieuses, CHU de Nice, France; i Service de Réanimation Médico-Chirurgicale, Hôpital Pasteur, Centre Hospitalier Universitaire de Nice, Nice, France; j UR2CA, Équipe CARRES Physiologie, Université Côte d’Azur, Nice, France

**Keywords:** Acute kidney injury, Endothelial dysfunction, Microcirculation, Renal repair.

## Abstract

**Background:**

The kidneys receive a disproportionately high share of cardiac output relative to their mass, reflecting their central role in solute clearance and fluid regulation. The renal microcirculation is anatomically complex and regionally heterogeneous, with steep gradients in perfusion and oxygen tension from cortex to medulla. This architecture supports efficient filtration and reabsorption but renders specific nephron segments supply-dependent and susceptible to injury during haemodynamic instability, hypoxia, inflammation, or exposure to toxins. These vulnerabilities can lead to acute kidney injury (AKI), where microvascular dysfunction often precedes changes in global renal perfusion.

**Main body:**

The renal microvasculature comprises glomerular capillaries followed by efferent arterioles supplying either cortical peritubular capillaries or the medullary vasa recta. Regional endothelial heterogeneity, limited medullary oxygen availability, countercurrent oxygen shunting, and the high metabolic demand of tubular sodium transport create intrinsic susceptibility to injury. In AKI, sepsis, ischaemia-reperfusion, surgery, nephrotoxins, and venous congestion may trigger early endothelial activation and glycocalyx injury, promoting increases permeability, leukocyte and platelet adhesion, altered vascular tone, capillary obstruction, and interstitial oedema. These changes impair perfused capillary density and oxygen diffusion. Microvascular dysfunction may persist despite normalisation of systemic haemodynamics, reflecting a loss of haemodynamic coherence and contributing to regional hypoxia and reduced glomerular filtration.

Tubular injury further amplifies these abnormalities. Reduced NaCl reabsorption increases delivery to the macula densa, activates tubuloglomerular feedback, and promotes afferent vasoconstriction, while tubular obstruction, filtrate backleak, and interstitial pressure oppose filtration and compromise peritubular perfusion. The relative contribution of these mechanisms differs across sepsis-associated, ischaemia-reperfusion, perioperative, nephrotoxic, congestive, and predominantly haemodynamic AKI phenotypes.

When repair is incomplete, persistent endothelial dysfunction, pericyte detachment, capillary rarefaction, chronic hypoxia, and fibrosis promote nephron dropout, loss of renal functional reserve, and progression from AKI to chronic kidney disease. Clinical assessment currently relies on complementary surrogates, including perfusion imaging, venous congestion assessment, oxygenation monitoring, and endothelial or tubular biomarkers.

**Conclusion:**

Renal microvascular dysfunction contributes to the initiation, functional expression, persistence, and long-term consequences of AKI. A phenotype-oriented approach integrating perfusion, congestion, oxygenation, endothelial injury, and tubulo-vascular interactions may improve mechanistic understanding and support the development of targeted preventive and therapeutic strategies.

## Introduction

Acute kidney injury (AKI) is one of the most frequent organ dysfunctions among critically ill patients and is associated with short- and long-term mortality and outcomes [1]. Depending on the population studied and the diagnostic criteria used, AKI may affect a substantial proportion of ICU patients and predisposes survivors to chronic kidney disease (CKD) [[1], [2], [3]]. Although the kidneys receive approximately 20% of cardiac output and filter 120–150 mL of plasma per minute, this abundant macrocirculatory supply does not guarantee homogeneous regional perfusion or oxygenation. The renal microcirculation is a critical interface through which circulating mediators and haemodynamic stressors may contribute to AKI. Its complex architecture, corticomedullary oxygen gradients, and coupling with tubular function also create areas of intrinsic vulnerability during haemodynamic instability, inflammation, hypoxia, congestion, or exposure to nephrotoxins.

AKI has been attributed mainly to renal hypoperfusion and ischaemia. However, renal macrocirculatory changes vary widely across experimental shock models. This variability depends on the type of injury (endotoxemia, sepsis, ischaemia-reperfusion, or hypovolemia), species studied, duration, timing, and methods [4]. While acknowledging that post-mortem sampling may itself introduce histological alterations, immediate post-mortem kidney biopsies from patients dying of septic shock have shown acute tubular lesions and prominent leukocyte infiltration, with apoptosis involving only a small proportion of tubular cells [5]. Experimental and clinical studies indicate that total renal blood flow during sepsis may be reduced, preserved, or increased and does not consistently correlate with the presence or severity of AKI [4,6,7]. These observations suggest that loss of kidney function cannot be explained solely by reduced global perfusion. Instead, AKI often reflects the interaction of altered vascular tone, increased endothelial permeability, microthrombi formation, inflammation, heightened changes in interstitial pressure, and tubular metabolic reprogramming [8].

AKI is not a single disease entity but a heterogeneous syndrome encompassing multiple clinical phenotypes, including sepsis-associated, ischaemia-reperfusion injuries (IRI), perioperative, nephrotoxic, congestive, and predominantly hemodynamic AKI. These phenotypes differ in their initiating insults, temporal profiles, and therapeutic implications. However, many of them converge on shared downstream mechanisms involving the renal microcirculation. In this sense, renal microvascular dysfunction should not be viewed as a universal or exclusive cause of AKI, but as a unifying framework through which diverse renal insults may lead to regional perfusion failure, impaired oxygen delivery, tubulo-vascular uncoupling, maladaptive repair, and progression towards CKD.

This review integrates renal haemodynamic, endothelial biology, regional oxygenation, tubulo-vascular crosstalk, and AKI-to-CKD transition into a translational perspective centred on the renal microcirculation. Following a mechanistic sequence, we first describe the physiological vulnerabilities of the renal microvascular network, then examine early endothelial and glycocalyx injury, functional microcirculatory failure, and maladaptive repair. We also discuss how these mechanisms vary across AKI phenotypes. Finally, we review emerging approaches for clinical assessment and evaluate therapeutic strategies.

## Search strategy and selection criteria

This article was designed as a narrative expert review rather than a systematic review. Relevant publications were identified through targeted searches of PubMed complemented by expert selection by the authors.

Articles were selected by the authors according to their relevance to the scope of the review, mechanistic or clinical importance, and contribution to the proposed integrative framework. For clinical interventions, randomised controlled trials, systematic reviews, and meta-analyses were prioritized whenever available. Experimental studies were included when they provided mechanistic insight not readily accessible in humans. No formal meta-analysis or risk-of-bias assessment was performed. Therefore, selection bias and publication bias cannot be excluded.

Throughout the manuscript, the nature of the supporting evidence is explicitly distinguished where relevant. We differentiate between renal experimental evidence derived from in vitro or in vivo kidney models, non-renal mechanistic evidence extrapolated from other vascular or cellular systems, human observational evidence, and interventional clinical evidence. Mechanistic conclusions based predominantly on experimental data are identified as such, and extrapolations from chronic kidney disease, diabetes, cardiovascular disease, or non-renal vascular biology are explicitly qualified. This distinction is descriptive and does not represent a formal grading of evidence.

## Renal microvascular architecture and physiological vulnerability

In healthy individuals, the kidneys receive 20% of the cardiac output, despite comprising less than 0.5% of total body weight. While the kidney also has one of the highest levels of oxygen consumption of any organ, global renal oxygen delivery substantially exceeds consumption, as blood flow is determined by the requirement for blood detoxification and body fluid homeostasis.

1 In-series organisation of the renal microcirculation

These functions demand a distinct serial arrangement of two capillary beds. Blood first traverses the glomerular capillaries, uniquely interposed between the afferent and efferent arterioles, before entering a second, postglomerular capillary network. In cortical nephrons, efferent arterioles form the dense cortical peritubular capillary (PTC) network surrounding the proximal and distal tubules, whereas those of juxtamedullary nephrons (representing approximately 15% of all nephrons) give rise to the descending and ascending vasa recta that accompany the long loops of Henle into the medulla and sustain exchange and the corticomedullary osmotic gradient (Fig. 1) [4,9]. Thus, the renal microcirculation is organised in series as a glomerular capillary bed followed by a regionally specialised postglomerular capillary bed, rather than as a homogeneous vascular network. This compartmentalised architecture creates marked regional differences in blood flow, oxygen delivery, and metabolic demand, thereby predisposing cortical and, particularly, outer medullary territories to distinct patterns of injury.

2 Regional perfusion gradients and oxygen shunting

**Fig. 1:**
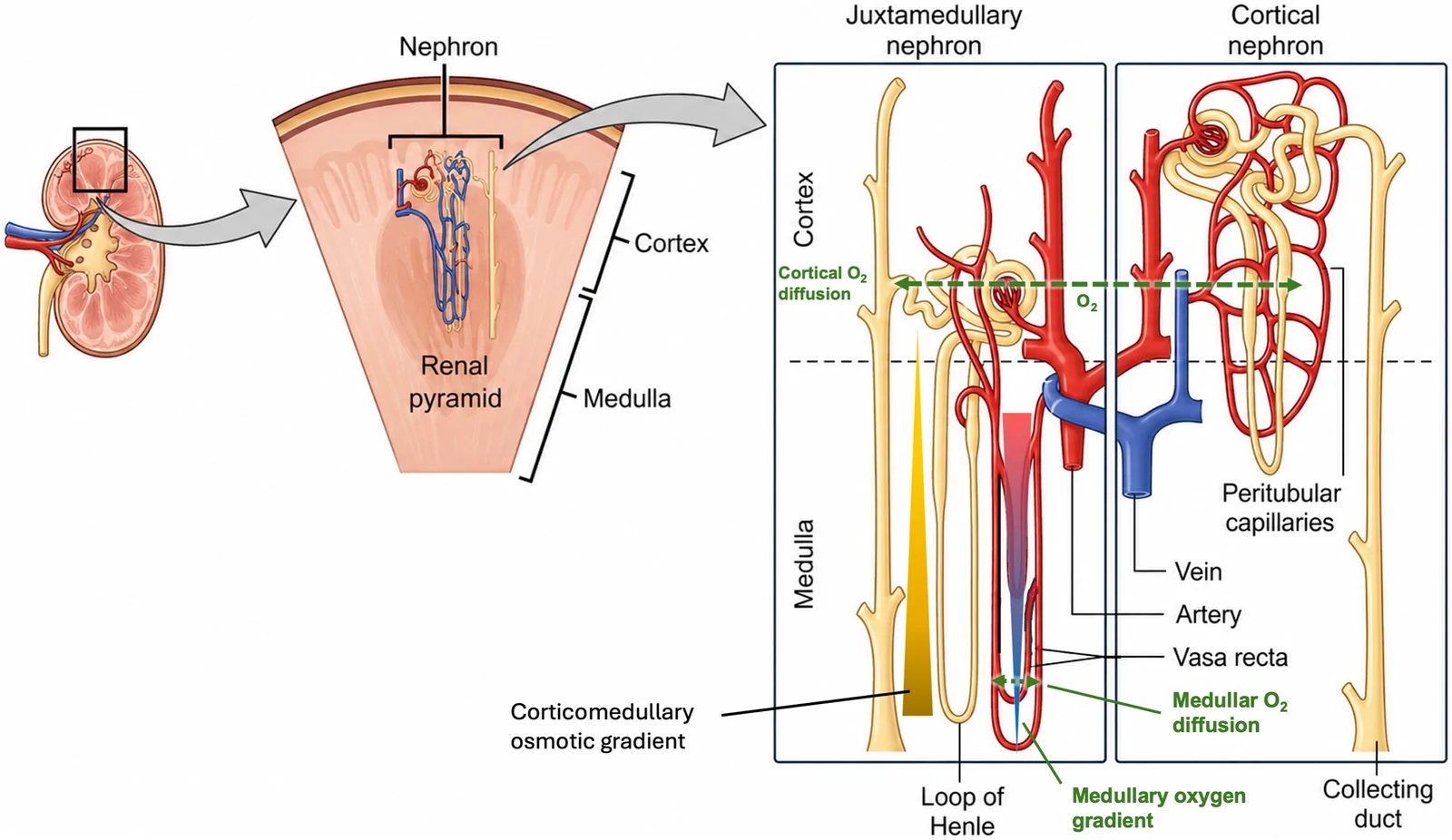
Regional organisation of cortical and juxtamedullary nephrons and their microvascular networks. The left-hand panels locate the nephron within the kidney and renal pyramid. The enlarged panels compare a juxtamedullary nephron, whose long loop of Henle extends deep into the medulla, with a cortical nephron, whose tubular segments remain predominantly within the cortex. The dashed line represents the corticomedullary junction.

The renal microvasculature has a complex flow distribution and regulatory mechanisms that enable organ function. This organisation enables high plasma ultrafiltration while allowing recovery of most filtered salt and water. More than 90% of the renal blood flow is distributed to the cortex with progressively lower perfusion as one descends into the hypertonic medulla, thereby limiting dissipation of the corticomedullary osmotic gradient. In juxtamedullary nephrons, descending vasa recta carry oxygenated blood from the corticomedullary junction into the medulla, while ascending vasa recta return blood towards the cortex and renal venous circulation. Their close, countercurrent arrangement permits oxygen to diffuse from descending to ascending vasa recta before reaching the deeper medulla, reducing oxygen delivery to medullary tissue despite preserved global renal blood flow. The loop structure of the vasa recta permits diffusional oxygen shunting (Fig. 1) [10]. Physiological and modelling studies indicate that a shunt may also occur in the cortex because of the close anatomical apposition of arterial inflow and venous outflow pathways, allowing oxygen to pass directly from arteries and arterioles to adjacent veins and venules and thereby bypass part of the capillary exchange surface [11]. Together, these perfusion gradients and intrarenal oxygen shunts contribute to the physiological hypoxia of the medulla and increase the susceptibility to further reductions in oxygen delivery or increases in tubular oxygen demand.

3 Supply-dependent areas and exposure to filtered inflammatory mediators and nephrotoxins.

Although mean oxygen consumption is lower in the medulla than in the cortex, the outer medulla and corticomedullary junction contain tubular segments with particularly high metabolic requirements. Active sodium reabsorption in these regions depends on basolateral Na^+^/K^+^-ATPase activity, especially in the S3 segment of the proximal tubule and the medullary thick ascending limb of the loop of Henle [12,13]. Their high oxygen demand, combined with limited regional perfusion and low tissue oxygen tension, places these segments close to the threshold of supply dependency. Consequently, even modest reductions in oxygen delivery or increases in tubular workload may produce local hypoxia despite apparently preserved global renal blood flow. Vulnerability is further increased by exposure to circulating inflammatory mediators and nephrotoxins, which may reach tubular epithelial cells through the blood flow or, when glomerularly filtered, through the tubular lumen. Experimental renal studies indicate that filtered inflammatory mediators may be taken up by proximal tubular cells or become concentrated along the nephron as water is reabsorbed, thereby amplifying epithelial exposure and injury

4 Renal endothelial cell heterogeneity

Renal endothelial cells (RECs) display marked phenotypic, transcriptional, and metabolic heterogeneity along the renal vascular tree, reflecting their anatomical location and functional coupling with surrounding epithelial structures (Fig. 2) [[14], [15], [16], [17], [18], [19], [20], [21]]. Single-cell transcriptomic studies have identified distinct endothelial populations along the renal vascular tree adapted to local mechanical forces, oxygen tension, osmotic conditions, and metabolic demands [14,21]. Although renal single-cell studies demonstrate metabolic heterogeneity of RECs, much of the mechanistic evidence linking glycolysis, fatty acid oxidation, mitochondrial metabolism, and redox homeostasis to endothelial function derives from broader endothelial biology [[16], [17], [18], [19],^22^].

**Fig. 2:**
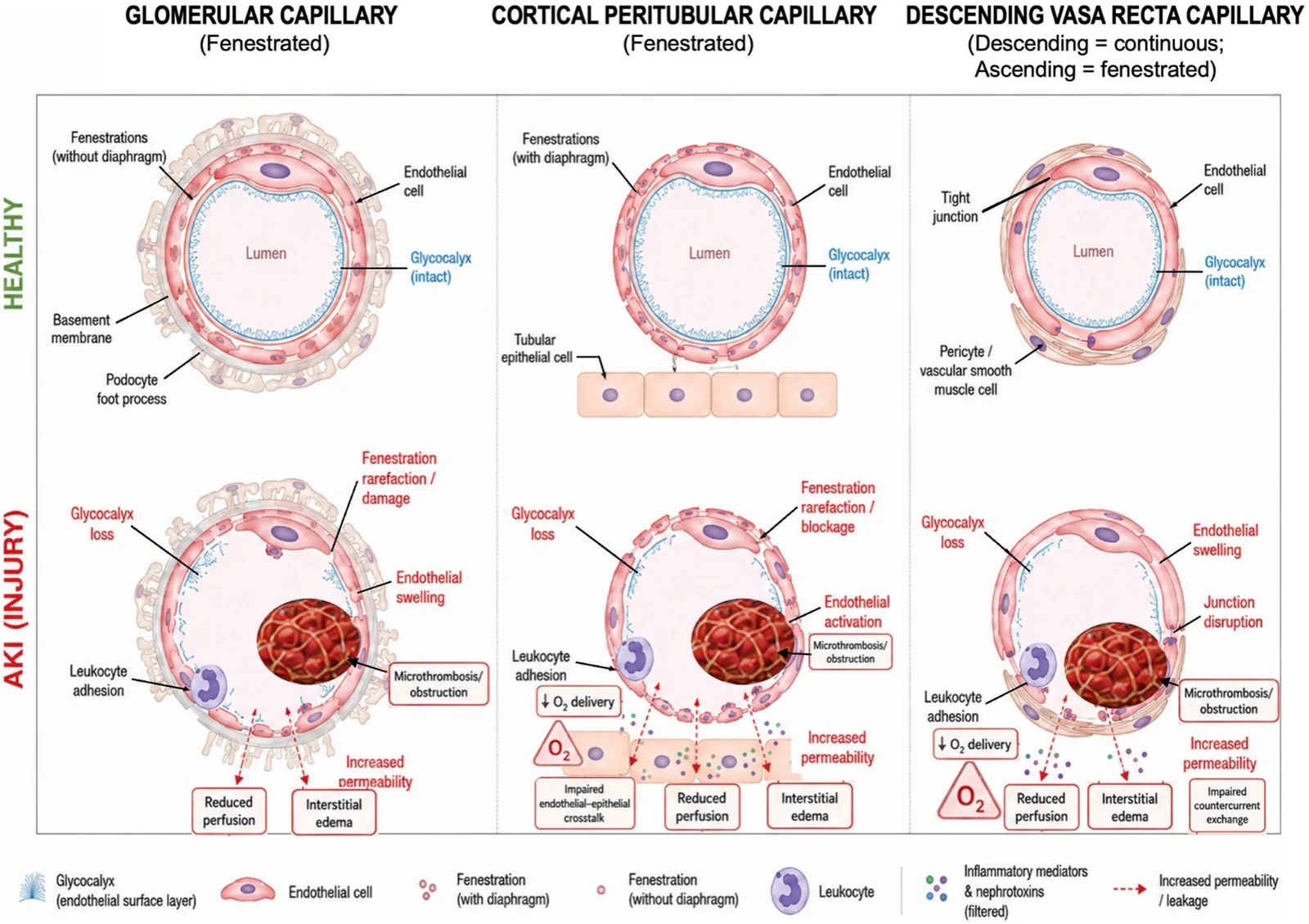
Regional heterogeneity of renal endothelial cells and compartment-specific alterations during acute kidney injury. Schematic cross-sectional representation of glomerular capillaries, cortical peritubular capillaries, and medullary descending vasa recta under healthy conditions (upper row) and during acute kidney injury (AKI; lower row). Descending vasa recta are lined by a continuous non-fenestrated endothelium (presented here), whereas ascending vasa recta have a thin fenestrated endothelium. Abbreviations: AKI, acute kidney injury; GBM, glomerular basement membrane.

### Glomerular endothelial cells

This heterogeneity is reflected across renal compartments. Glomerular endothelial cells are highly fenestrated and contribute, together with the glycocalyx, glomerular basement membrane, and podocytes, to the filtration barrier [20,23]. Cortical peritubular endothelial cells are fenestrated and closely coupled to tubular transport, with experimental studies identifying subpopulations involved in metabolic regulation, angiogenesis, and immune surveillance [14,16,21]. Medullary RECs display specialised transcriptional adaptations to the hypoxic and hyperosmolar medullary environment. These regional phenotypes may influence susceptibility to sustained hypoxia and impaired vascular repair, although their contribution to human AKI remains incompletely established.

### Cortical peritubular endothelial cells

Cortical PTCs are composed of fenestrated RECs functionally coupled to proximal and distal tubular segments, facilitating the exchange of water, ions, and solutes [16]. Distinct cortical endothelial subpopulations have been identified, including cells enriched in lipid metabolism pathways and expressing natriuretic peptides involved in sodium and volume regulation. Experimental data also suggest the presence of endothelial subsets with angiogenic or immune surveillance functions, although their relevance in human AKI remains to be established [14,21].

### Medullary renal endothelial cells

Medullary RECs exhibit specialised transcriptional activity that reflects the extreme osmotic and hypoxic environments of the renal medulla. These cells regulate urea transport, water permeability, and osmolarity-responsive pathways that are essential for urine concentration [16]. Endothelial gene expression varies along the cortico-papillary axis, supporting fine-tuned local vascular responses but conferring high susceptibility to sustained hypoxia and microvascular rarefaction during AKI [14]. This regional specialisation may contribute to vulnerability to sustained hypoxia; its contribution to capillary rarefaction during human AKI remains incompletely established.

## Early endothelial dysfunction and structural microvascular injury in AKI

Inflammation plays a central role in AKI-induced microcirculatory dysfunction. Inflammatory mediators, including DAMPs and PAMPs, are released into the circulation during sepsis, trauma, ischaemia, or surgical injuries. Interactions with injured tubular cells stimulate the production of cytokines and adhesion molecules [24]. These mediators, such as tumour necrosis factor-alpha (TNF-α), interleukin-6 (IL-6), and interleukin-1 beta (IL-1β), promote endothelial activation, leukocyte recruitment, and increased microvascular permeability (Fig. 2) [25]. Experimental renal studies support that interactions between DAMPs/PAMPs and tubular epithelial cells stimulate the production of reactive oxygen species (ROS), resulting in exacerbated oxidative stress, mitochondrial injury, and apoptosis [26].

For clarity, endothelial dysfunction, structural microvascular injury, and capillary rarefaction represent related but distinct stages of renal microvascular involvement in AKI. Endothelial dysfunction refers primarily to potentially reversible functional alterations, including abnormal vasoreactivity, reduced NO bioavailability, increased permeability, leukocyte and platelet adhesion, and heterogeneous capillary perfusion, without necessarily implying loss of vascular structure. Structural microvascular injury refers to physical disruption of the endothelial barrier and vascular architecture, including glycocalyx degradation, endothelial cell injury, loss of intercellular junction integrity, basement membrane alterations, or endothelial cell loss. By contrast, capillary rarefaction is reserved here for the persistent anatomical loss of peritubular capillaries or vasa recta resulting from failed vascular repair. Thus, reduced perfused capillary density during acute AKI should not be considered synonymous with structural capillary rarefaction.

### Endothelial activation and glycocalyx degradation

Renal endothelial activation is an early response to inflammatory, hypoxic, nephrotoxic, and haemodynamic insults. Mechanistic studies in vascular endothelium and experimental renal models indicate that these stimuli promote a proinflammatory and pro-oxidant phenotype characterised by reduced nitric oxide (NO) bioavailability, altered cellular metabolism, increased expression of adhesion molecules, and impaired vascular reactivity [[27], [28], [29], [30], [31], [32]]. These abnormalities may compromise microvascular homeostasis and endothelial repair, particularly in sepsis-associated, IRI, and perioperative AKI [33].

Endothelial and tubular injury are closely interconnected. Reduced capillary perfusion promotes local hypoxia and oxidative stress, whereas injured tubular epithelial cells release cytokines, ROS, and other mediators capable of further activating adjacent endothelial cells. Experimental renal studies therefore support bidirectional endothelial–tubular crosstalk as an amplifier of early kidney injury.

### Glycocalyx injury and barrier disruption

Evidence from experimental AKI and systemic endothelial studies indicates that endothelial activation may be accompanied by glycocalyx shedding, disruption of intercellular junctions, increased permeability, and leukocyte adhesion [34]. Capillary leakage and interstitial oedema increase oxygen diffusion distances and may further impair regional perfusion. These mechanisms have been demonstrated predominantly in experimental renal and microvascular models.

Endothelial barrier stability is also regulated by the angiopoietin–Tie2 pathway. Human observational studies have associated angiopoietin–Tie2 imbalance with severe sepsis-associated and perioperative AKI [35,36], whereas experimental activation of Tie2 preserves renal endothelial integrity and attenuates ischaemia-reperfusion AKI [37,38].

### Venous congestion and interstitial oedema

Venous congestion is an important determinant of renal interstitial oedema and impaired microvascular perfusion. Clinically, renal venous congestion may result from right ventricular dysfunction, fluid overload, positive-pressure mechanical ventilation, or intra-abdominal hypertension. Experimental increases in renal venous pressure and human observational studies of congestion support a mechanism whereby increased renal venous pressure reduces the effective arteriovenous pressure gradient and is transmitted to the renal microvasculature and interstitium. Within the relatively non-compliant renal capsule, the resulting increase in renal interstitial hydrostatic pressure can compress PTCs, restrict capillary inflow, and further impair regional oxygen delivery [39,40]. Increased interstitial pressure is, in turn, transmitted to the tubule, increasing intratubular pressure and opposing glomerular ultrafiltration. Thus, renal congestion may reduce both renal perfusion and glomerular filtration even when arterial pressure is preserved.

Given their sensitivity, changes may be more localized to areas of oxygen supply dependence (S3 segment of the proximal tubule and thick ascending limb of the loop of Henlé). This regional pattern is commonly observed after experimental IRI and during systemic inflammation associated with sepsis, trauma, or major surgery. It may partly explain the apparent dissociation between relatively focal tubular structural injury and a profound reduction in kidney function [41]. Conversely, direct nephrotoxic injury from drugs or pigment nephropathy is typically more evenly distributed along the proximal tubule.

### Leukocyte adhesion, platelet activation, and microthrombosis

Experimental AKI studies demonstrate that endothelial activation and reduced blood-flow velocity are accompanied by platelet activation, microthrombus formation, and capillary plugging [42]. This leads to patterns of heterogeneous microvascular perfusion across capillary networks, thus commonly observed “failure of microvascular reperfusion” despite restoration of arterial inflow reflecting a loss of haemodynamic coherence. Intrarenal shunting may further reduce effective tissue perfusion [7,43]. Platelet activation, leukocyte adhesion, and focal microvascular thrombosis may contribute to capillary obstruction and heterogeneous perfusion in experimental AKI. Human histopathological studies in sepsis-associated AKI have reported fibrin deposition and occasional vascular thrombosis, but findings are heterogeneous and do not support microthrombosis as a universal feature of human AKI [44]. At the extreme end of the spectrum, extensive intravascular thrombosis may cause renal cortical necrosis (Table 1), particularly after prolonged global ischaemia or in severe endothelial disorders such as thrombotic microangiopathy. More commonly, regional loss of perfused capillary density aggravates hypoxia and oxidative stress in the corticomedullary junction and outer medulla, where tubular segments are especially vulnerable to impaired oxygen delivery.

**Table 1:** Compartment-specific acute microvascular alterations, functional consequences, and maladaptive repair after acute kidney injury.

Renal compartment and physiological role	Acute microvascular alterations	Functional consequences	Maladaptive repair and long-term consequences	Evidence base and limitations
Glomerular microcirculation Afferent arteriole-glomerular capillary-efferent arteriole axis controlling glomerular pressure and filtration-barrier function.	Altered afferent and efferent arteriolar tone; Endothelial swelling; Glycocalyx degradation; Loss of fenestrations; Leukocyte and platelet plugging; Focal microthrombosis.	Reduced glomerular capillary pressure or effective filtration surface; Reduced GFR; Impaired filtration-barrier selectivity.	Basement membrane remodelling; Glomerular capillary dropout; Glomerulosclerosis; Compensatory hyperfiltration in surviving nephrons; Nephron loss and reduced renal functional reserve.	Physiological and experimental evidence, with supporting clinical haemodynamic data [20,23,[69], [70], [71], [72], [73]]. Persistent glomerular structural changes and loss of renal functional reserve are supported by experimental and longitudinal clinical evidence [76,83,84].
Cortical peritubular capillaries Support cortical oxygen delivery, tubular reabsorption, secretion, and solute exchange.	Endothelial activation; Glycocalyx loss; Leukocyte and platelet adhesion; Microvascular plugging; Heterogeneous perfusion and altered flow distribution; Capillary leakage; Interstitial oedema and capillary compression.	Reduced oxygen delivery and increased diffusion distance; Cortical tubular dysfunction; Impaired reabsorption and secretion; Interstitial oedema; Amplification of GFR reduction through tubuloglomerular feedback.	Peritubular capillary rarefaction; Basement membrane thickening; Pericyte detachment; Extracellular matrix deposition; Tubular atrophy; Interstitial fibrosis and nephron dropout.	Predominantly experimental evidence. Acute endothelial permeability, capillary plugging, and perfusion heterogeneity [30,34,41,42]; pericyte detachment, rarefaction, and fibrosis [32,[78], [79], [80], [81], [82]]. Direct human evidence remains limited.
Vasa recta and outer-medullary microcirculation Maintain medullary oxygen delivery, countercurrent exchange, and the corticomedullary osmotic gradient.	Endothelial and glycocalyx injury; Capillary plugging; Reduced or heterogeneous flow; Altered intrarenal redistribution and oxygen shunting; Interstitial oedema.	Outer-medullary hypoxia; Injury or dysfunction of the S3 proximal tubule and medullary thick ascending limb; Impaired sodium transport and urinary concentrating ability.	Potential vasa recta rarefaction; Persistent medullary hypoxia; Tubulointerstitial fibrosis; Sustained concentrating defect and loss of renal reserve.	Predominantly physiological and experimental evidence for regional perfusion, oxygen shunting, and outer-medullary hypoxia [[9], [10], [11], [12], [13],^41^,^58^,^59^,^74^,^75^]. Persistent medullary rarefaction is mainly experimental; direct human evidence remains limited [66,77,79,82].
Tubulo-vascular unit Integrative functional unit comprising tubular epithelial cells, adjacent peritubular capillary or vasa recta endothelial cells, pericytes, and the intervening interstitium.	Loss of endothelial-epithelial coupling; Cytokine and ROS-mediated bidirectional injury; Tubular transport failure; Capillary and tubular leakage; Obstruction and filtrate backleak; Oedema and capillary compression; Pathological tubuloglomerular feedback.	Increased tubular back-pressure; Reduced GFR; Impaired tubular reabsorption and secretion; Worsening regional hypoxia and interstitial oedema.	Persistent epithelial cell-cycle arrest; Pericyte-to-myofibroblast transition; Capillary rarefaction; Tubular atrophy; Interstitial fibrosis; Nephron dropout and loss of renal functional reserve.	Predominantly experimental evidence, with limited human observational support. Acute transport failure, tubuloglomerular feedback, and backleak [8,12,41,69]; maladaptive repair, mitochondrial dysfunction, cell-cycle arrest, and fibrosis [64,[66], [67], [68],^76^,^81^,^83^,^84^].

GFR, glomerular filtration rate; ROS, reactive oxygen species.

Evidence descriptors indicate the predominant source and translational maturity of the supporting evidence and do not represent a formal grading of certainty.

### Altered vascular tone and NO dysregulation

Under physiological conditions, endothelial NO synthase (eNOS)-derived NO supports endothelial homeostasis, vasodilation, and regional perfusion. During AKI, endothelial dysfunction reduces NO bioavailability due to impaired synthesis via eNOS and arginase activity. The loss of this vasodilatory signal favors vasoconstriction, thereby reducing perfusion, which worsens renal hypoperfusion and limits oxygen delivery [45]. The mechanisms involved in the reduction of eNOS activity have not been fully identified. Experimental renal IRI studies provide direct evidence that depletion of tetrahydrobiopterin (BH4), an essential eNOS cofactor, is associated with loss of functional eNOS dimerization, reduced NO production, and eNOS uncoupling [46,47]. In this uncoupled state, electron transfer through eNOS is no longer efficiently coupled to NO synthesis and instead favours superoxide generation, further amplifying oxidative stress and endothelial dysfunction [46,48]. This creates a self-reinforcing cycle of reduced NO bioavailability and increased ROS production that can impair renal microvascular perfusion. Importantly, this mechanistic sequence is supported predominantly by experimental IRI models and endothelial cell studies and has not been directly demonstrated across all forms of human AKI. These mechanisms have been described across a range of pathological settings, including disease-specific conditions and chronic exposures. They may help explain previous observations linking preoperative microcirculatory dysfunction to adverse postoperative outcomes [[49], [50], [51], [52]]. The resulting endothelial dysfunction shifts vascular tone away from endothelium-dependent vasodilation towards exaggerated vasoconstriction. Importantly, experimental studies indicate that eNOS inhibition worsens ischaemic injury, whereas increased eNOS expression may attenuate sepsis-induced AKI [53]. Additionally, studies have indicated that local eNOS inhibition and increased inducible NO synthase (iNOS) activity may contribute to dysregulated vasodilation and oxidative stress, whereas local NO deficiency may persist in other microvascular territories, contributing to heterogeneous renal perfusion and impaired renal function [54]. Experimental septic models have shown increased cortical but reduced medullary NOS expression, a pattern that may favour cortical vasodilation while contributing to medullary hypoperfusion and hypoxia [6]. These abnormalities impair autoregulation and contribute to heterogeneous regional perfusion rather than producing uniform renal vasoconstriction. Both afferent and efferent arteriolar vasodilation may occur, and a proportionally greater reduction in efferent arteriolar resistance has been proposed to decrease glomerular capillary hydrostatic pressure and GFR despite preserved or increased total renal blood flow.

## Functional consequences of microcirculatory failure

The microcirculation serves as an essential interface between the bloodstream and parenchymal cells [55]. The primary function of the microcirculation is to ensure the effective exchange of oxygen, nutrients, metabolic waste, and signalling molecules across tissues. Beyond passive exchanges, microcirculation actively regulates tissue perfusion through dynamic control of vascular tone and capillary recruitment, thereby maintaining homeostasis and supporting organ function. This regulation depends on the interplay between convective forces (driven by cardiac output and pressure gradients) and diffusive forces (influenced by the distance between capillaries, surrounding extracellular matrix, and target cells). Microvascular endothelial cells respond to a wide array of stimuli, including shear stress, metabolic changes, and inflammatory signals, which modulate vasomotor tone and permeability [56].

1 Loss of haemodynamic coherence

Systemic haemodynamic variables provide only an indirect estimate of renal microvascular perfusion. Mean arterial pressure, cardiac output, and total renal blood flow may normalize after resuscitation while regional capillary perfusion remains heterogeneous or reduced. This mismatch between restored macrocirculatory variables and persistent microcirculatory dysfunction represents a loss of haemodynamic coherence, sometimes described as macro–microcirculatory dissociation [57].

In the kidney, loss of haemodynamic coherence may result from persistent endothelial dysfunction, capillary obstruction, altered arteriolar reactivity, venous congestion, interstitial oedema, or intrarenal shunting. Consequently, restoration of arterial pressure or global renal blood flow does not necessarily restore regional tissue perfusion, oxygen delivery, or glomerular filtration. This phenomenon is particularly relevant in sepsis-associated and perioperative AKI, in which correction of systemic haemodynamic abnormalities may occur without simultaneous recovery of the renal microcirculation [57]. Importantly, these functional abnormalities may markedly reduce perfused capillary density and tissue oxygen delivery without implying anatomical loss of the capillary network and may therefore remain at least partly reversible if the underlying haemodynamic and inflammatory disturbances resolve. This concept derives largely from experimental and clinical studies of shock and systemic microcirculation, with renal evidence remaining predominantly physiological and observational.

2 Impaired oxygen delivery and regional hypoxia

Renal hypoxia, driven by microcirculatory dysfunction, is a key feature of AKI. It may result from four complementary mechanisms: reduced convective oxygen delivery due to decreased cardiac output or renal perfusion; increased diffusion distance caused by interstitial oedema, capillary rarefaction, or capillary-tubular separation; diffusional oxygen shunting between closely apposed renal vessels (Fig. 1); and venous congestion, which reduces the effective arteriovenous pressure gradient and increases renal interstitial pressure [11,39,40]. These mechanisms may coexist and impair tissue oxygenation even when systemic haemodynamic variables and total renal blood flow appear adequate [8,58,59]. In experimental sepsis-associated AKI, impaired renal oxygen utilisation is accompanied by alterations in tubular mitochondrial function and metabolic reprogramming, which may contribute to reduced renal function [60]. Regional heterogeneity further contributes to oxygen imbalance: experimental sepsis models demonstrate that cortical perfusion and oxygenation may remain relatively preserved despite progressive medullary hypoperfusion and hypoxia, reflecting altered intrarenal vascular resistance, heterogeneous capillary recruitment, and oxygen shunting [4,58,59]. These effects are particularly relevant in the outer medulla, where the S3 proximal tubular segment and medullary thick ascending limb have high transport-related oxygen requirements but limited oxygen reserve [11]. Moreover, in experimental septic AKI, restoration of arterial pressure with norepinephrine further reduced medullary perfusion and oxygen tension despite preserved cortical perfusion and oxygenation, suggesting that oxygen shunting contributes to kidney hypoxia [58].

3 Oxidative stress and mitochondrial dysfunction

Renal hypoxia and oxidative stress are closely interconnected during AKI. In experimental ischaemia–reperfusion AKI, mitochondrial ROS promote mitochondrial DNA damage, impaired mitochondrial function, inflammatory signalling, and renal injury, whereas suppression of mitochondrial ROS attenuates these abnormalities [61]. In experimental sepsis-associated AKI, tubular mitochondrial dysfunction and ROS generation may also occur despite preserved global renal oxygen delivery, indicating that oxidative stress is not solely a consequence of tissue hypoperfusion [62]. Together, these findings support a feed-forward interaction between oxidative stress, mitochondrial dysfunction, inflammation, and cellular injury without extensive apoptosis or necrosis [63].

Metabolic reprogramming during AKI is best characterised in tubular epithelial cells. In experimental sepsis-associated AKI, proximal tubular cells exhibit altered mitochondrial respiration, reduced ATP availability, impaired fatty acid oxidation, and changes in glycolytic capacity [60]. Adaptive responses, including mitophagy and cell-cycle arrest, may favour cellular survival while reducing tubular transport and renal function [64]. By contrast, metabolic adaptation of renal endothelial cells is less directly characterised: single-cell studies demonstrate marked segment-specific metabolic specialisation, but its causal contribution to AKI remains predominantly supported by experimental evidence [14,16]. Although these mechanisms initially protect cells from apoptosis, they contribute to cellular desquamation with potential increased permeability resulting in the leakage of glomerular filtrate from the tubular lumen to the interstitium, leading to interstitial kidney oedema [65].

Persistent microcirculatory dysfunction perpetuates renal tissue hypoxia, initiating and maintaining a cycle of progressive injury characterised by cellular stress, inflammation, and fibrosis, ultimately hindering AKI recovery. Hypoxia activates hypoxia-inducible factors (HIFs), which regulate genes involved in angiogenesis, cellular energy metabolism (such as glycolysis and mitochondrial respiration), and profibrogenic processes, thereby exacerbating renal fibrosis and promoting chronic kidney disease progression [66]. Additionally, mitochondrial dysfunction, specifically abnormal mitophagy, compromises cellular recovery, intensifying susceptibility to prolonged tubular injury, as demonstrated in IRI and sepsis models, in which disrupted mitochondrial homeostasis perpetuates severe renal damage [67,68].

4 Tubuloglomerular feedback and preglomerular vasoconstriction

Experimental and physiological studies support enhanced tubuloglomerular feedback as one contributor in AKI [4]. Tubular dysfunction can, in turn, amplify microvascular and glomerular impairment. Reduced sodium reabsorption in the proximal tubule and thick ascending limb increases NaCl delivery to the macula densa. This activates the tubuloglomerular feedback mechanism in response to the loss of tubular reabsorption of chloride in the proximal tubule, leading to a higher chloride concentration in the distal tubule (Fig. 3). This is sensed in the macula densa, causing preglomerular vasoconstriction via a paracrine adenosine A1 receptor-mediated response [69]. The reduction in glomerular capillary hydrostatic pressure, despite preserved total renal blood flow, results in diminished glomerular filtration (Table 1).

**Fig. 3:**
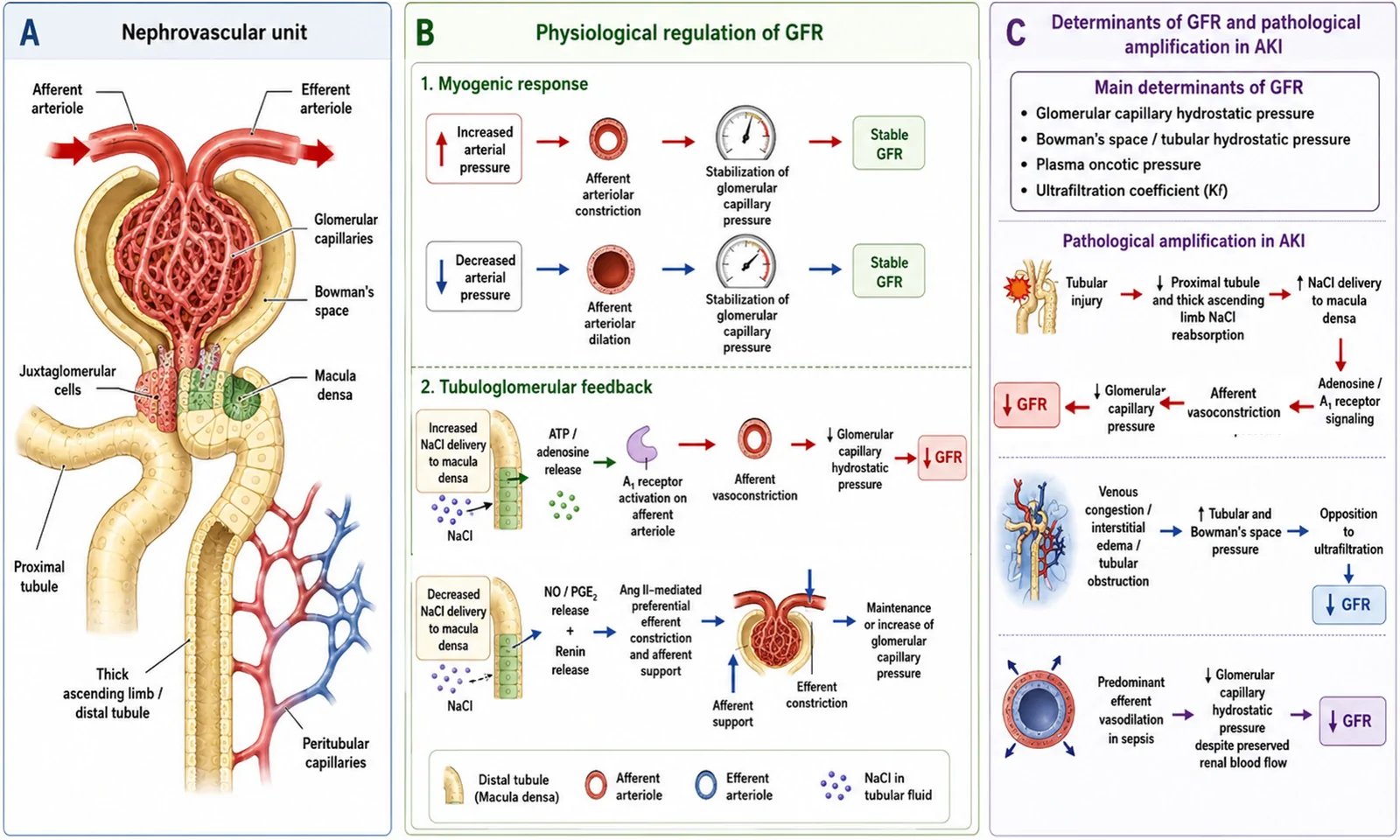
Regulation of glomerular filtration rate and tubuloglomerular feedback. (A) Schematic representation of the nephrovascular unit. (B) Physiological regulation of glomerular filtration rate (GFR) by the myogenic response and the tubuloglomerular feedback. (C) Mechanisms contributing to GFR reduction during AKI. Abbreviations: AKI, acute kidney injury; ATP, adenosine triphosphate; GFR, glomerular filtration rate; NaCl, sodium chloride; NO, nitric oxide; PGE₂, prostaglandin E₂.

In more severe injury, experimental AKI models also support contributions from tubular obstruction, filtrate backleak and increased intratubular pressure, and interstitial oedema. Thus, tubular dysfunction is translated into glomerular filtration failure through the combined effects of afferent vasoconstriction and increased downstream pressure. While this afferent vasoconstriction is reversible in principle, in reality, resolution of inflammation and regeneration of functional tubular epithelial cells are required to restore glomerular ultrafiltration and nephron function.

5 Impaired autoregulation and glomerular pressure

Under physiological conditions, renal autoregulation maintains renal blood flow and GFR despite fluctuations in systemic blood pressure. This regulation depends on the myogenic response of the afferent arteriole, thereby limiting transmission of systemic pressure fluctuations to the glomerulus (Fig. 3) [70]. The second mechanism involved is the tubuloglomerular feedback, through which changes in NaCl delivery to the macula densa modify afferent arteriolar tone [4].

Experimental studies and observational studies in shock suggest impaired renal autoregulation during AKI (Table 1) [71,72]. Indirect human observational data suggest that systemic or cutaneous microvascular dysfunction, defined as dysregulated, delayed, and excessive vasodilatation, which could lead to poor tolerance of arterial pressure lability, may pre-exist, at least in part, the renal insult [49].

This distinction is particularly relevant in sepsis-associated AKI. Both afferent and efferent arteriolar vasodilation may occur, with a proportionally greater fall in efferent resistance in some experimental models [73]. The resulting reduction in glomerular capillary hydrostatic pressure may markedly decrease GFR despite preserved or even increased total renal blood flow. Tubular dysfunction may further contribute by increasing NaCl delivery to the macula densa and activating tubuloglomerular feedback, thereby reinforcing afferent vasoconstriction and filtration decline. Thus, the fall in GFR during sepsis may reflect altered intrarenal resistance distribution and glomerulotubular signalling rather than a uniform reduction in renal perfusion.

6 Reduced GFR

A reduction in GFR is a defining functional feature of AKI but does not necessarily imply a proportional decrease in total renal blood flow. Glomerular filtration depends on the balance between glomerular capillary hydrostatic pressure, pressure within Bowman’s space and the tubular lumen, plasma oncotic pressure, and the glomerular ultrafiltration coefficient (Fig. 3). Alterations in any of these determinants may therefore reduce GFR.

Changes in the relative tone of the afferent and efferent arterioles can lower glomerular capillary pressure despite preserved whole-kidney perfusion. Experimental studies suggest that endothelial swelling and filtration-barrier injury may additionally reduce the effective ultrafiltration coefficient.

Downstream pressure also contributes to filtration failure. Venous congestion increases renal interstitial and PTC pressures, while interstitial oedema and tubular obstruction increase intratubular and Bowman’s space hydrostatic pressure, thereby opposing ultrafiltration. Impaired tubular sodium reabsorption increases NaCl delivery to the macula densa, activates the tubuloglomerular feedback, and promotes afferent arteriolar vasoconstriction. Finally, the fall in GFR during AKI therefore reflects the combined effects of altered glomerular pressure, increased tubular back-pressure, congestion, and tubuloglomerular feedback rather than reduced renal perfusion alone.

7 Phenotype-specific patterns

AKI should not be considered a single microvascular phenotype. Similar clinical manifestations, including oliguria and reduced GFR, may result from different combinations of impaired arterial inflow, altered intrarenal vascular resistance, endothelial and tubular injury, venous congestion, and inflammatory activation. In sepsis-associated AKI, inflammatory endothelial dysfunction and heterogeneous peritubular flow may coexist with preserved or increased total renal blood flow. Afferent and efferent arteriolar vasodilation, intrarenal blood flow redistribution, and capillary flow heterogeneity may reduce glomerular filtration pressure and medullary oxygenation despite preserved cortical or whole-kidney perfusion [59]. By contrast, ischaemia–reperfusion AKI is characterised by an initial interruption of oxygen delivery followed by oxidative and inflammatory reperfusion injury, endothelial swelling, capillary obstruction, and persistent microvascular no-reflow after restoration of arterial inflow reflecting a loss of haemodynamic coherence [74,75]. Other AKI contexts produce distinct but overlapping patterns. Perioperative AKI commonly combines transient hypotension or low cardiac output with inflammation, haemodilution, venous congestion, embolic or thrombotic events, and nephrotoxic exposure. Nephrotoxic AKI is predominantly initiated by segment-specific tubular exposure and injury, with secondary endothelial activation and peritubular microvascular dysfunction. Congestive AKI is driven mainly by elevated renal venous and interstitial pressures, whereas predominantly haemodynamic AKI results from reduced renal perfusion pressure or altered afferent–efferent arteriolar tone, with potentially limited structural injury when promptly reversed. These patterns are neither mutually exclusive nor temporally stable, and their relative contributions may vary within individual patients over time. A predominantly functional and potentially reversible haemodynamic or endothelial disturbance may resolve without structural damage, whereas persistent or severe insults may progress to endothelial and microvascular structural injury. Failure of subsequent vascular repair may ultimately result in true capillary rarefaction and maladaptive remodelling. Their main features and potential clinical implications are summarised in Table 2.

**Table 2:** Phenotype-specific microvascular patterns in acute kidney injury.

AKI phenotype	Dominant trigger	Main microvascular abnormalities	Oxygenation and perfusion pattern	Potential clinical implications / management considerations	Evidence base and limitations
Sepsis-associated AKI	Dysregulated host response with circulating PAMPs/DAMPs and cytokines	Glycocalyx shedding; heterogeneous capillary flow; leukocyte and platelet adhesion; focal microthrombosis may occur; altered NO signalling; afferent and efferent vasodilation, sometimes with a greater reduction in efferent resistance	Total renal blood flow may be reduced, preserved, or increased; capillary flow may be hyperdynamic, sluggish, intermittent, or absent; cortical perfusion may remain relatively preserved while medullary oxygenation decreases	Source control and antimicrobial therapy; individualised fluid and vasopressor therapy; avoidance of fluid overload and additional nephrotoxins	Predominantly experimental evidence, with supporting human physiological and observational data. Direct human renal microcirculatory measurements remain limited, and mechanisms vary over time [4,6,7,25,43,44,58,59].
Ischaemia-reperfusion AKI	Abrupt interruption or severe reduction of renal blood and oxygen delivery followed by reperfusion	Endothelial swelling; glycocalyx shedding; ROS generation; leukocyte and platelet plugging; heterogeneous capillary perfusion and microvascular no-reflow; cortical necrosis may occur in severe or prolonged injury	Initial global hypoperfusion followed by persistent heterogeneous capillary perfusion and outer medullary hypoxia despite restored arterial inflow	Minimise ischaemic duration; restore flow and oxygen delivery promptly; avoid recurrent hypotension, hypoxaemia, and additional nephrotoxic insults. Remote ischaemic preconditioning remains investigational because clinical results are inconsistent.	Predominantly experimental evidence; direct translation to human AKI is limited because ischaemia–reperfusion incompletely reproduces most clinical phenotypes [41,71,74,75].
Perioperative AKI	Combined haemodynamic, inflammatory, embolic, and nephrotoxic insults	Endothelial activation; heterogeneous capillary perfusion; impaired autoregulation; haemodilution-related reduction in oxygen content; venous congestion; occasional microembolisation or microthrombosis	Variable combination of reduced convective oxygen delivery, loss of haemodynamic coherence, inflammatory microvascular dysfunction, and medullary oxygen supply–demand mismatch	Individualised haemodynamic and oxygen-delivery optimisation; avoidance of prolonged hypotension, excessive haemodilution, fluid overload, and nephrotoxic exposure; use of established perioperative kidney-protection bundles where appropriate.	Mixed experimental and human observational evidence. Mechanisms vary by surgical setting, and direct human renal microcirculatory evidence remains limited [33,49,57].
Nephrotoxic AKI	Filtered, secreted, accumulated, or metabolised drugs; contrast agents; haemoglobin or myoglobin pigments; environmental toxins	Segment-specific tubular epithelial injury, commonly involving the proximal tubule; oxidative and mitochondrial stress; tubular obstruction; secondary inflammation and peritubular endothelial dysfunction	Regional oxygen-demand mismatch may develop, but primary global renal hypoperfusion is not required; distribution depends on the toxin and tubular transport pathway	Stop or reduce the causative exposure; adjust dose; use toxin-specific hydration, antidotes, or extracorporeal removal when indicated	Toxin-specific experimental and clinical evidence. Avoidance or dose adjustment is clinically established; the contribution of primary microvascular injury varies.
Congestive AKI	Elevated central and renal venous pressure, right ventricular failure, fluid overload, or increased intra-abdominal pressure	Reduced renal arteriovenous pressure gradient; increased peritubular capillary and interstitial pressure; interstitial oedema; capillary compression; increased tubular back-pressure	Renal blood flow may be relatively preserved, but effective perfusion pressure, filtration, and tissue oxygen diffusion are impaired	Assess venous congestion clinically and, where appropriate, with adjunctive Doppler-based tools such as VExUS. Individualise decongestion and treatment of right-sided or global cardiac dysfunction.	Physiological and human observational evidence. VExUS may aid assessment, but treatment thresholds and outcome benefit from VExUS-guided decongestion remain unproven [39,40,86].
Predominantly haemodynamic AKI	Hypovolaemia, low cardiac output, systemic vasodilation, hypotension, or drugs altering afferent or efferent arteriolar tone	Reduced renal perfusion pressure; autoregulatory failure; afferent vasoconstriction or excessive efferent vasodilation; initially limited endothelial or tubular structural injury	Total renal flow and filtration decrease together; reduced tubular sodium delivery lowers oxygen consumption and may partially limit hypoxic mismatch	Correct the underlying abnormality in circulating volume, arterial pressure, or cardiac output using fluids, vasopressors, or inotropes as clinically appropriate.	Physiological and clinical haemodynamic evidence; direct renal microcirculatory evidence is limited. Persistent AKI suggests superimposed structural or inflammatory injury [[70], [71], [72]].

AKI, acute kidney injury; DAMPs, damage-associated molecular patterns; NO, nitric oxide; PAMPs, pathogen-associated molecular patterns; ROS, reactive oxygen species; VExUS, venous excess ultrasound score.

Evidence descriptors indicate the predominant source and translational maturity of the supporting evidence and do not represent a formal grading of certainty.

## Maladaptive repair and long-term structural consequences

Recovery of serum creatinine or GFR after AKI does not necessarily imply complete structural recovery. Whereas early endothelial dysfunction and reductions in perfused capillary density may be reversible, persistent structural injury can impair vascular regeneration and ultimately lead to true capillary rarefaction, chronic hypoxia, fibrosis, and nephron loss (Table 1) [76]. This sequence is well supported by experimental AKI studies, whereas human longitudinal evidence primarily establishes the association between AKI and subsequent CKD without directly demonstrating all intermediate microvascular mechanisms.

1 Persistent endothelial dysfunction and failed vascular repair

In established and persistent AKI, acute microvascular injury may evolve into incomplete vascular repair and persistent structural abnormalities (Table 1) [76,77]. Experimental studies have shown that impaired endothelial proliferation and angiogenic responses can limit restoration of the PTC network after AKI [32,78,79]. Altered signalling between endothelial cells, tubular epithelial cells, and pericytes may further compromise vascular repair and capillary stability [77]. Persistent barrier dysfunction and abnormal vascular recovery can thereby sustain regional hypoxia and inflammation, providing an early mechanistic link between AKI and subsequent chronic structural damage.

2 Pericyte detachment, microvascular rarefaction and chronic hypoxia

Pericyte detachment is an important component of failed microvascular repair. Experimental studies have shown that loss of pericyte-endothelial contact after AKI destabilizes PTCs and can promote subsequent capillary rarefaction [80]. Detached pericytes may also migrate into the interstitium and acquire a matrix-producing myofibroblast phenotype, thereby linking microvascular rarefaction directly to fibrogenesis [81].

A key structural consequence of failed vascular repair is microvascular rarefaction, characterised by permanent loss of PTCs and, potentially, vasa recta rather than restoration of the pre-injury vascular network [77]. Experimental studies demonstrate that PTC density and perfusion may remain reduced long after apparent recovery from AKI [80]. The resulting increase in capillary-to-tubule diffusion distance and reduction in regional oxygen delivery favour chronic tissue hypoxia, which may further impair tubular repair and promote progressive tubulointerstitial injury.

3 Myofibroblast transformation and fibrosis

The transition of activated pericytes towards a myofibroblast phenotype provides a direct connection between microvascular injury and extracellular matrix deposition. Experimental lineage-tracing and signalling studies have shown that renal pericytes contribute substantially to the population of matrix-producing myofibroblasts after kidney injury [81]. Progressive extracellular matrix accumulation and basement membrane remodelling can further narrow or obliterate PTCs, reinforcing microvascular rarefaction and establishing a self-perpetuating cycle of hypoxia, inflammation, and fibrosis [77].

Experimental studies indicate context-dependent effects of hypoxia-inducible factor (HIF) signalling in this process. Transient HIF activation may support cellular adaptation and repair, whereas persistent or dysregulated hypoxic signalling can contribute to inflammatory and profibrotic gene expression. Together with maladaptive tubular cell-cycle arrest, mitochondrial dysfunction, and persistent immune activation, these changes drive tubulointerstitial scarring and the AKI-to-CKD transition (Table 1) [66].

4 Glomerular alterations, nephron dropout, and loss of renal functional reserve

Although post-AKI remodelling predominantly affects the tubulointerstitial and peritubular compartments, glomerular and arteriolar abnormalities may also persist. Experimental studies have described endothelial injury, loss of capillary loops and fenestrations, basement membrane remodelling, and progressive glomerulosclerosis months after ischaemia-reperfusion AKI [83]. Progressive tubular atrophy and nephron loss may futher reduce the number of functioning nephrons and increase the filtration burden placed on those that remain.

Apparent recovery of serum creatinine may therefore overestimate true renal recovery. Reduced creatinine generation caused by critical illness-associated muscle loss can mask persistent impairment, while compensatory increases in single-nephron GFR may maintain whole-kidney filtration despite a reduced nephron number. This compensatory hyperfiltration occurs at the expense of renal functional reserve and may expose the remaining glomeruli to sustained haemodynamic stress. Consequently, patients may have a near-normal estimated GFR but limited capacity to respond to subsequent physiological or pathological demands. Loss of renal functional reserve, chronic tubulointerstitial hypoxia, and glomerular hyperfiltration collectively increase susceptibility to recurrent AKI and progressive CKD. Repeated episodes of injury may therefore establish a self-reinforcing cycle of nephron loss, declining reserve, and increasing vulnerability to further insults [84].

## Clinical assessment of renal microcirculatory dysfunction

Direct assessment of the renal microcirculation remains challenging because of the deep location of the kidney, the marked regional heterogeneity of perfusion and oxygenation, and the limited accessibility of the medulla.

1 Perfusion imaging

Contrast-enhanced ultrasound (CEUS) is a bedside, non-invasive imaging tool that uses gas-filled microbubbles as intravascular tracers to assess real-time macro- and microcirculatory changes. CEUS provides dynamic visualization of renal cortical perfusion. In septic or ischaemic AKI, it reveals cortical hypoperfusion, even when the global renal blood flow appears normal or increased on Doppler [85]. In practice, CEUS predominantly assesses cortical rather than the medullary perfusion. Its clinical use remains limited by operator and acquisition dependence, variability in image analysis, and the absence of standardized diagnostic or therapeutic thresholds.

2 Venous congestion and pressure-related assessment

Elevated central or renal venous pressure and intra-abdominal hypertension reduce the effective renal arteriovenous pressure gradient and increase interstitial pressure. The resulting interstitial oedema may compress PTCs, while transmission of pressure to the tubular compartment increases tubular backpressure and opposes glomerular filtration.

Intrarenal venous Doppler can identify progressive discontinuity of venous flow associated with renal congestion. The venous excess ultrasound score (VExUS), which combines inferior vena cava size with hepatic, portal, and intrarenal venous Doppler patterns, provides a broader assessment of systemic venous congestion [86]. These techniques may complement clinical examination when deciding whether further fluid administration or decongestion is appropriate. However, they do not directly measure renal microvascular perfusion, validated treatment thresholds are lacking, and outcome benefits from VExUS-guided decongestion have not been established.

3 Oxygenation monitoring

Beyond microvascular flow, oxygen measurements have emerged as promising AKI markers. Urinary oxygen pressure (PuO₂) is a reliable surrogate of renal medullary oxygenation and can be used as a real-time biomarker of acute renal stress in cardiac, laparoscopic, and open abdominal surgery [[87], [88], [89]]. However, it does not directly measure renal tissue oxygen tension and may be influenced by urine flow, temperature, and perioperative factors affecting oxygen consumption. Emerging data also suggest the value of mitochondrial oxygen tension as an early indicator of hypoxia. Using the delayed fluorescence of protoporphyrin IX after 5-aminolevulinic acid priming, two pilot studies showed that a mitochondrial oxygen tension <30 mmHg at ICU admission and the cumulative time below this threshold were associated with AKI before an increase in lactate in patients who underwent cardiac surgery [90,91].

4 Biomarkers

Circulating biomarkers may provide indirect evidence of the biological processes underlying renal microvascular dysfunction. Syndecan-1 reflects glycocalyx shedding, whereas angiopoietin-2, soluble thrombomodulin, and soluble Tie2 have been evaluated as markers of endothelial activation or injury. Inflammatory and coagulation-related markers, including IL-6, D-dimers, and platelet activation markers, may indicate a proinflammatory or prothrombotic environment but are not specific to the renal microcirculation [35].

Urinary or plasma NGAL and urinary KIM-1 indicate tubular injury, whereas urinary TIMP-2 and IGFBP7 reflect tubular cell stress and identify patients at increased short-term risk of moderate-to-severe AKI [92,93]. These biomarkers may complement functional criteria such as serum creatinine and urine output, but none directly quantifies renal microvascular perfusion. Their interpretation is limited by variable kinetics, dependence on the clinical context, imperfect renal specificity, and the absence of biomarker-specific therapeutic targets.

## Strategies targeting the renal microcirculation in AKI

Current management in AKI therefore remains based on correction of the underlying cause, optimisation of systemic haemodynamics and venous outflow, and avoidance of additional renal insults. Overall, the certainty of evidence supporting a specific effect of these interventions on the renal microcirculation remains predominantly experimental or observational, with limited interventional clinical evidence. Although renal microvascular dysfunction represents a potential therapeutic target, no intervention has yet been shown to improve patient-centred outcomes.

1 Strategies to optimising renal perfusion

Restoring adequate arterial pressure and cardiac output is essential, but normalisation of systemic haemodynamic variables does not necessarily restore renal microvascular perfusion because loss of haemodynamic coherence may persist [57]. Fluid administration, vasopressors, inotropes, and decongestive strategies should therefore be individualised according to the dominant haemodynamic phenotype, avoiding both arterial underfilling and venous congestion. CEUS may detect changes in predominantly cortical perfusion that are not apparent from systemic variables or conventional Doppler measurements [85]. However, it remains primarily a research and phenotyping tool, and no evidence currently supports CEUS-guided fluid or vasoactive therapy [94,95].

An alternative approach to preserving perioperative renal perfusion is the recruitment of renal functional reserve. In the PROTECTION trial, 3511 adults undergoing cardiac surgery with cardiopulmonary bypass were randomised to receive a continuous intravenous infusion of a balanced amino acid mixture (2 g/kg of ideal body weight per day for up to 3 days) or a placebo [96]. Amino acid infusion reduced the incidence of creatinine-defined AKI from 31.7% to 26.9% (relative risk 0.85; 95% CI, 0.77–0.94; P = 0.002), with a reduction in stage 3 AKI from 3.0% to 1.6% (relative risk 0.56; 95% CI, 0.35–0.87). The physiological rationale for this intervention is that amino acid loading increases renal blood flow and glomerular filtration, thereby recruiting renal functional reserve [97]. This response is thought to reflect amino acid-induced changes in intrarenal haemodynamics rather than the pharmacological effect of a single amino acid. Importantly, PROTECTION evaluated a balanced amino acid mixture and was not designed to test a specific amino acid-dependent mechanism. Moreover, renal microvascular perfusion was not directly assessed, and the extent to which recruitment of renal functional reserve translates into protection from structural kidney injury remains uncertain.

2 Modulating vascular tone

### Vasopressors

Vasopressor therapy may improve systemic pressure and global renal perfusion while producing variable effects on regional oxygenation. In some experimental settings, increased arterial pressure has not restored—and may aggravate—medullary hypoxia despite preserved renal blood flow or renal venous oxygen content. This dissociation highlights that renal oxygenation depends not only on total oxygen delivery but also on its intrarenal distribution, diffusion to tubular cells, venous outflow, and local oxygen shunting.

### Vasodilators (Nitric Oxide Donors, Prostacyclin Analogues)

Experimental manipulation of nitric oxide pathways can improve renal microvascular flow, but clinical evidence remains limited. Administration of NO donors, such as sodium nitroprusside or L-arginine, improves cortical perfusion and attenuates tubular necrosis in animal models [98,99]. In a clinical trial, inhaled NO administered during cardiopulmonary bypass through the oxygenator and for six hours postoperatively significantly reduced the incidence of AKI, with a relative risk of 0.59 (95% CI, 0.35 to 0.99; P = 0.043) [100]. However, it remains unclear whether this effect is primarily due to improved right ventricular function and oxygenation without systemic vasodilation and lower venous congestion or a direct action on the renal microcirculation.

Iloprost is a prostacyclin analogue that plays an important role in maintaining microcirculatory renal blood flow and augmenting tissue perfusion. Prostacyclin analogues exert vasodilatory and antiplatelet effects while preserving endothelial barrier function. In preclinical IRI models, prostacyclins improved renal capillary flow and reduced leukocyte adhesion and cytokine expression [101,102]. However, in two randomised clinical trials involving patients with septic shock, continuous infusion of iloprost did not reduce or mitigate the incidence or severity of AKI, particularly when assessed using changes in the renal component of the SOFA score [103,104].

### Agents that reduce vasoconstriction (endothelin receptor antagonists)

Endothelin-1 (ET-1), a potent vasoconstrictor, is markedly upregulated in AKI and contributes to both preglomerular vasoconstriction and PTC rarefaction [105]. Selective antagonism of the ET-A receptor improves renal blood flow, reduces inflammatory infiltration, and prevents fibrosis in preclinical models [106]. Interestingly, these benefits were not observed with dual endothelin-A/B receptor antagonists. Most current investigations focus on evaluating endothelin receptor antagonists in CKD, particularly their ability to significantly reduce proteinuria and enhance GFR [107].

3 Preserving endothelial barrier function

Strategies aimed at preserving or restoring endothelial barrier integrity remain predominantly experimental in AKI. Sulodexide has reduced glycocalyx shedding and vascular permeability in rodent AKI models, while angiopoietin-1 and Tie2 agonists and sphingosine-1-phosphate pathway modulation have shown barrier-stabilising effects in experimental settings. In patients with septic shock, a post hoc analysis of the ADRENAL trial associated hydrocortisone with a lower requirement for new kidney replacement therapy [108], but this does not establish a glycocalyx-mediated mechanism. No endothelial barrier-targeted intervention has yet demonstrated clinical efficacy specifically through improvement of the renal microcirculation.

4 Limiting microvascular obstruction

Relatively few studies (experimental and clinical) have explored the therapeutic potential of antiplatelet or anticoagulant strategies to prevent or mitigate AKI in septic or perioperative contexts [44]. Observational studies investigating the role of aspirin, which showed a benefit in renal dysfunction, were cardiac surgery studies [109,110]. However, this benefit was offset in noncardiac surgery studies [111,112]. The balance between efficacy and bleeding risk, particularly in critically ill and perioperative patients, limits its widespread adoption.

One experimental agent of mechanistic interest is dual antiplatelet and anticoagulant (APAC), a mast cell heparin proteoglycan mimetic with potent dual antiplatelet and anticoagulant properties. In various vascular injury models, APAC has been shown to effectively inhibit thrombosis. Notably, in a rat model of renal IRI, APAC conferred renoprotective effects even in moderate-to-severe IRI by attenuating vascular damage and suppressing innate immune activation [113].

5 Targeting inflammation and oxidative stress

Antioxidants, such as N-acetylcysteine (NAC), vitamins B1, C, and E, and melatonin, have demonstrated renoprotective effects in animal models by scavenging ROS, preserving mitochondrial function, and maintaining PTCs integrity [114]. However, the clinical efficacy of NAC in preventing AKI has been conflicting [115]. Furthermore, meta-analyses of randomised clinical trials in patients with sepsis evaluating high-dose vitamin C or resuscitation cocktails aimed at reducing oxidative stress have failed to show any significant clinical benefits, particularly regarding renal function [116,117].

Alkaline phosphatase (ALP), a detoxifying agent, has also been proposed as a potential therapy for sepsis-associated AKI owing to its protective effects against lipopolysaccharide-induced inflammation and renal injury in animal models [118]. However, clinical studies with recombinant human ALP have not confirmed these findings; in patients with sepsis-associated AKI, ALP administration failed to produce a significant improvement in kidney function, as assessed by creatinine clearance on day 7 [119].

6 Hemoadsorption and blood purification

Extracorporeal blood purification strategies, such as hemoadsorption, have demonstrated benefits in preclinical models by removing pro-inflammatory mediators, including TNF-α, IL-1, IL-6, alarmins, and other DAMPs, thereby reducing renal inflammation and improving microvascular perfusion. However, meta-analyses of clinical studies have produced divergent results and have not specifically assessed outcomes related to kidney function [120,121]. The use of extracorporeal blood purification with the oXiris membrane has also yielded inconsistent results in the prevention of cardiac surgery-associated AKI [122,123]. Robust data from randomised controlled trials on AKI are still lacking.

7 Targeting metabolic adaptation and repair

Pharmacological modulation of cellular metabolism remains an experimental therapeutic concept in AKI. Metformin has shown protective effects in experimental ischaemic, nephrotoxic, and sepsis-associated AKI through pathways involving AMP-activated protein kinase (AMPK) activation, mitochondrial homeostasis, and autophagy [[124], [125], [126]]. sodium–glucose cotransporter-2 (SGLT2) inhibitors and glucagon-like peptide-1 (GLP-1) receptor agonists also influence cellular energy metabolism, autophagy, and oxidative stress, but most supporting clinical evidence derives from diabetes, cardiovascular disease, or chronic kidney disease [[127], [128], [129], [130]]. Direct evidence in established AKI remains sparse, and efficacy as a treatment for acute AKI has not been established in randomised clinical trials [129,131,132]. These approaches should therefore remain considered hypothesis-generating in AKI and require dedicated safety and efficacy trials.

## Conclusion

Renal microvascular and endothelial dysfunction provide an important integrative framework for understanding the initiation, persistence, and recovery of several AKI phenotypes, but they do not account for all forms of AKI. The regional heterogeneity of renal perfusion and oxygenation, together with the close coupling between glomerular, peritubular, and tubular function, makes the kidney particularly vulnerable to disturbances in vascular tone, capillary flow, venous pressure, inflammation, and oxygen delivery.

This contribution appears particularly important in severe or persistent AKI, notably in sepsis-associated and ischaemia-reperfusion AKI. In these settings, endothelial activation, glycocalyx injury, capillary flow heterogeneity, microvascular obstruction, interstitial oedema, and altered tubulo-glomerular crosstalk may sustain regional hypoxia and reduced filtration despite restoration of systemic haemodynamics, thereby producing a loss of haemodynamic coherence. Microvascular dysfunction should therefore be considered alongside global perfusion, tubular injury, and glomerular pressure when interpreting the clinical expression and recovery of AKI.

Current methods for assessing renal microcirculatory dysfunction remain indirect and incompletely validated. Similarly, interventions aimed at preserving endothelial integrity, modulating vascular tone, reducing capillary obstruction, or supporting metabolic recovery are biologically plausible but have not yet demonstrated consistent patient-centred benefit. Future studies should integrate phenotype-oriented assessment, microvascular-related endpoints, and conventional renal outcomes to determine whether targeted monitoring and treatment can improve recovery and reduce progression from AKI to CKD.

## Authors’ contributions

S. Abrard: Conceptualisation, Writing original draft, Visualisation, Supervision.

J. Prowle: Writing original draft, Visualisation.

P. Guerci: Writing original draft.

D. Payen: Writing review & editing, Visualisation.

T. Rimmelé: Conceptualisation, Methodology, Writing review & editing, Supervision, Project administration.

## Declaration of Generative AI and AI-assisted technologies in the writing process

The authors used ChatGPT (OpenAI) to assist with figure creation and English-language editing during the preparation of this manuscript. All AI-assisted outputs were verified and edited by the authors, who take full responsibility for the accuracy and integrity of the final manuscript and figures.

## Funding

None.

## Declaration of competing interest

Stanislas Abrard has received honoraria for consulting from Viatris Inc. (France) and from Public Health Expertise (France). Thomas Rimmelé has received honoraria for lectures at scientific conferences from Vantive and Fresenius Medical Care.
